# Simultaneous determination of volatile phenol, cyanide, anionic surfactant, and ammonia nitrogen in drinking water by a continuous flow analyzer

**DOI:** 10.1038/s41598-023-28776-w

**Published:** 2023-02-01

**Authors:** Guofu Qin, Keting Zou, Fengrui He, Ji Shao, Bei Zuo, Jia Liu, Ruixiao Liu, Bixia Yang, Guipeng Zhao

**Affiliations:** 1 Xi’an Center for Disease Control and Prevention, Xi’an, 710054 China; 2 Hangzhou Occupational Disease Prevention and Control Hospital, Hangzhou, 310014 China

**Keywords:** Environmental sciences, Chemistry

## Abstract

This study developed a method for the simultaneous determination of volatile phenol, cyanide, anionic surfactant, and ammonia nitrogen in drinking water, using a continuous flow analyzer. The samples were first distilled at 145 °C. The phenol in the distillate then subsequently reacted with alkaline ferricyanide and 4-aminoantipyrine to form a red complex that was measured colorimetrically at 505 nm. Cyanide in the distillate subsequently reacted with chloramine T to form cyanogen chloride, which then formed a blue complex with pyridinecarboxylic acid that was measured colorimetrically at 630 nm. The anionic surfactant reacted with basic methylene blue to form a compound that was extracted into chloroform and washed with acidic methylene blue to remove interfering substances. The blue compound in chloroform was determined colorimetrically at 660 nm. Ammonia reacted with salicylate and chlorine from dichloroisocyanuric acid to produce indophenol blue at 37 °C in an alkaline environment that was measured at 660 nm. The relative standard deviations were 0.75–6.10% and 0.36–5.41%, respectively, and the recoveries were 96.2–103.6% and 96.0–102.4% when the mass concentration of volatile phenol and cyanide was in the range of 2–100 μg/L. The linear correlation coefficients were ≥ 0.9999, and the detection limits were1.2 μg/L and 0.9 μg/L, respectively. The relative standard deviations were 0.27–4.86% and 0.33–5.39%, and the recoveries were 93.7–107.0% and 94.4–101.7%. When the mass concentration of anionic surfactant and ammonia nitrogen was 10–1000 μg/L. The linear correlation coefficients were 0.9995 and 0.9999, and the detection limits were 10.7 μg/L and 7.3 μg/L, respectively. When compared to the national standard method, no statistically significant difference was found. This approach saves time and labor, has a lower detection limit, higher precision and accuracy, less contamination, and is more appropriate for the analysis and determination of large-volume samples.

## Introduction

The markers of organoleptic, physical, and nonmetal elements in drinking water are volatile phenol, cyanide, anionic surfactant, and ammonia nitrogen^1^. Phenolic compounds are essential chemical building blocks with numerous uses, but phenol and its homologs are also poisonous and cannot be easily biodegraded. They are released during many industrial production processes and have emerged as common environmental contaminants^2,3^. Highly toxic phenolic substances can be absorbed into the body through the skin and respiratory system. Most lose their toxicity after entering the human body during detoxification processes and are then eliminated in the urine. However, when the amount surpasses the body's normal capacity for detoxification, excess components might build up in various organs and tissues, leading to chronic poisoning, headaches, rashes, skin pruritus, mental anxiety, anemia, and a variety of neurological symptoms^4–7^. Cyanide is extremely harmful but is common in nature. Numerous foods and plants contain cyanide, which can be created by specific bacteria, fungi, or algae^8,9^. In rinse-off products like shampoos and body washes, anionic surfactants are frequently utilized to assist in cleaning because they give these products the exceptional foaming and lathering qualities that consumers seek. However, many surfactants irritate the skin^10,11^. The drinking, ground, surface water, and wastewater contains nitrogen in the form of free ammonia (NH_3_) and ammonium salts (NH_4_^+^), which is referred to as ammonia nitrogen (NH_3_-N). The decomposition products of nitrogen-containing organic matter in domestic sewage by microbes, primarily from industrial effluents like coking and synthetic ammonia, account for some of the ammonia nitrogen in water^12–14^. Numerous methods, including spectrophotometry^15–17^, chromatography^18–21^ and flow injection^15,22–24^ be used to measure these four contaminants in water. Compared with other approaches, spectrophotometry is by far the most popular^1^. In this investigation, four dual-channel modules were employed to simultaneously assess volatile phenol, cyanide, anionic surfactants, and sulfide.

## Materials and methods

### Instruments and reagents

An AA500 continuous flow analyzer (SEAL, Germany), anSL252 electronic balance (Shanghai Mingqiao Electronic Instrument Factory, China), and a Milli-Q ultrapure water meter (Merck Millipore, USA) were used. All chemicals used in this work were analyticalgrade, and deionized water was used in all experiments. Hydrochloric acid, sulfuric acid, phosphoric acid, boric acid, chloroform, ethanol, sodium tetraborate, isonicotinic acid, and 4-aminoantipyrine were purchased from Sinopharm Chemical Reagent Co., Ltd. (China). Triton X-100, sodium hydroxide, and potassium chloride were obtained from Tianjin Damao Chemical Reagent Factory (China). Potassium ferricyanide, sodium nitroprusside, sodium salicylate, and *N*, *N*-dimethylformamide were supplied by Tianjin Tianli Chemical Reagent Co., Ltd. (China). Potassium dihydrogen phosphate, disodium hydrogen phosphate, pyrazolone, and methylene blue trihydratewere prepared from Tianjin Kemiou Chemical Reagent Co., Ltd. (China). Trisodium citrate dihydrate, polyoxyethylene lauryl ether, and sodium dichloroisocyanurate were purchased from Shanghai Aladdin Biochemical Technology Co., Ltd. (China). The standard solutions of volatile phenol, cyanide, anionic surfactant, and ammonia nitrogen in water were purchased from the China Academy of Metrology.

## Experimental procedures

### Volatile phenol reagent

Distillation reagent: dilute 160 mL phosphoric acid to 1000 mL with deionized water. Reserve buffer: weigh 9 g boric acid, 5 g sodium hydroxide, and 10 g potassium chloride and dilute them to 1000 mL with deionized water. Absorption reagent (updated every week): accurately measure 200 mL of reserve buffer, add 1 mL of 50% Triton X-100 (v/v, Triton X-100/ethanol), and use it after filtering through a 0.45 µm filter membrane. Potassium ferricyanide (updated weekly): weigh 0.15 g of potassium ferricyanide and dissolve it in 200 mL reserve buffer, add 1 mL of 50% Triton X-100, and use it after filtering through a 0.45 µm filter membrane. 4-aminoantipyrine (updated weekly): weigh 0.2 g of 4-aminoantipyrine and dissolve it in 200 mL reserve buffer, add 1 mL of 50% Triton X-100, and use it after filtering through a 0.45 µm filter membrane.

### Cyanide reagent

Distillation reagent: volatile phenol. Buffer: weigh 3 g of potassium dihydrogen phosphate, 15 g of disodium hydrogen phosphate, and 3 g of trisodium citrate dihydrate in deionized water and dilute to 1000 mL. Then, add 2 mL of 50% Triton X-100. Chloramine T: weigh 0.2 g chloramine T and dilute it to 200 mL with deionized water. Chromogenic agent: chromogenic agent A: completely dissolve 1.5 g of pyrazolone in 20 mL of *N*, *N*-dimethylformamide. Developer B: dissolve 3.5 gisonicotinic acid and 6 mL of 5 M NaOH in 100 mL deionized water. Before use, mix chromogenic agent A and developer B, adjust the pH to 7.0 with NaOH solution or HCl solution, then dilute to 200 mL with deionized water, and filter before use.

### Anionic surfactant reagent

Stock buffer: Dissolve 10 g sodium tetraborate and 2 g sodium hydroxide in deionized water and dilute to 1000 mL. 0.025% methylene blue solution: dissolve 0.05 g methylene blue trihydrate in deionized water and dilute to 200 mL. Methylene blue stock buffer (updated every day): dilute 20 mL of 0.025% methylene blue solution to 100 mL with the stock buffer. Transfer to a separatory funnel and wash with 20 mL chloroform, discard the used chloroform, and wash again with fresh chloroform until no red is observed in the chloroform layer (usually 3 times), and then filter. Alkaline methylene blue: dilute 60 mL of the filtered methylene blue stock buffer to 200 mL with the stock buffer, add 20 mL of ethanol, mix evenly, and degas. Acid methylene blue: add 2 mL of 0.025% methylene blue solution to about 150 mL of deionized water, add 1.0 mL of 1% H_2_SO_4_, and then add it to 200 mL with deionized water. Then, add 80 mL of ethanol, mix evenly, and degas.

### Ammonia nitrogen reagent

20% polyoxyethylene lauryl ether solution: Weigh 20 g polyoxyethylene lauryl ether and dilute it to 1000 mL with deionized water. Buffer: weigh 20 g trisodium citrate, dilute to 500 mL with deionized water, and add 1.0 mL of 20% polyoxyethylene lauryl ether. Sodium salicylate solution (updated every week): weigh 20 g sodium salicylate and 0.5 g potassium nitrite ferricyanide, and dissolve them in 500 mL deionized water. Sodium dichloroisocyanurate solution (updated every week): weigh 10 g sodium hydroxide and 1.5 g sodium dichloroisocyanurate, and dissolve them in 500 mL deionized water.

### Preparation of standard series

Volatile phenol and cyanide standards were formulated into 0 μg/L, 2 μg/L, 5 μg/L, 10 μg/L, 25 μg/L, 50 μg/L, 75 μg/L, and 100 μg/L solutions using 0.01 M sodium hydroxide solution. Anionic surfactants and ammonia nitrogen standards were prepared using deionized water to prepare 0 μg/L, 10 μg/L, 50 μg/L, 100 μg/L, 250 μg/L, 500 μg/L, 750 μg/L, and 1000 μg/L solutions.

### Analysis and detection

Start the cooling circulation tank, then (in order) turn on the computer, the sampler, and the power of the AA500 host, check whether the pipeline is connected correctly, press the air hose into the air valve, cover the peristaltic pump pressure plate, and put the reagent pipeline into pure water. Start the software, and activate the corresponding channel window, check whether each connecting tube is soundly connected and if there is looseness or leakage. If there is no liquid leakage, draw the corresponding reagent. After the baseline of the channel window stabilizes, select and run the set method file for detection and analysis. The instrument conditions are shown in Table 1.

**Table 1:** Instrument conditions.

Analyte	Distillation temperature (℃)	Heating pool temperature (℃)	Sample time (S)	Wash time (S)	Gain/AUFS	Sample /h	Sample to wash	Colorimetric wavelength (nm)
Cyanide	145	60	80	100	150/0.067	20	0.8	630
Volatile phenol	155	/	80	100	200/0.050	20	0.8	505
Anionic surfactant	/	/	80	100	60/0.167	20	0.8	660
Ammonia nitrogen	/	37	80	100	50/0.200	20	0.8	660

"/" means room temperature, and there is no temperature requirement.

## Results

### Method principle

**Figure 1:**
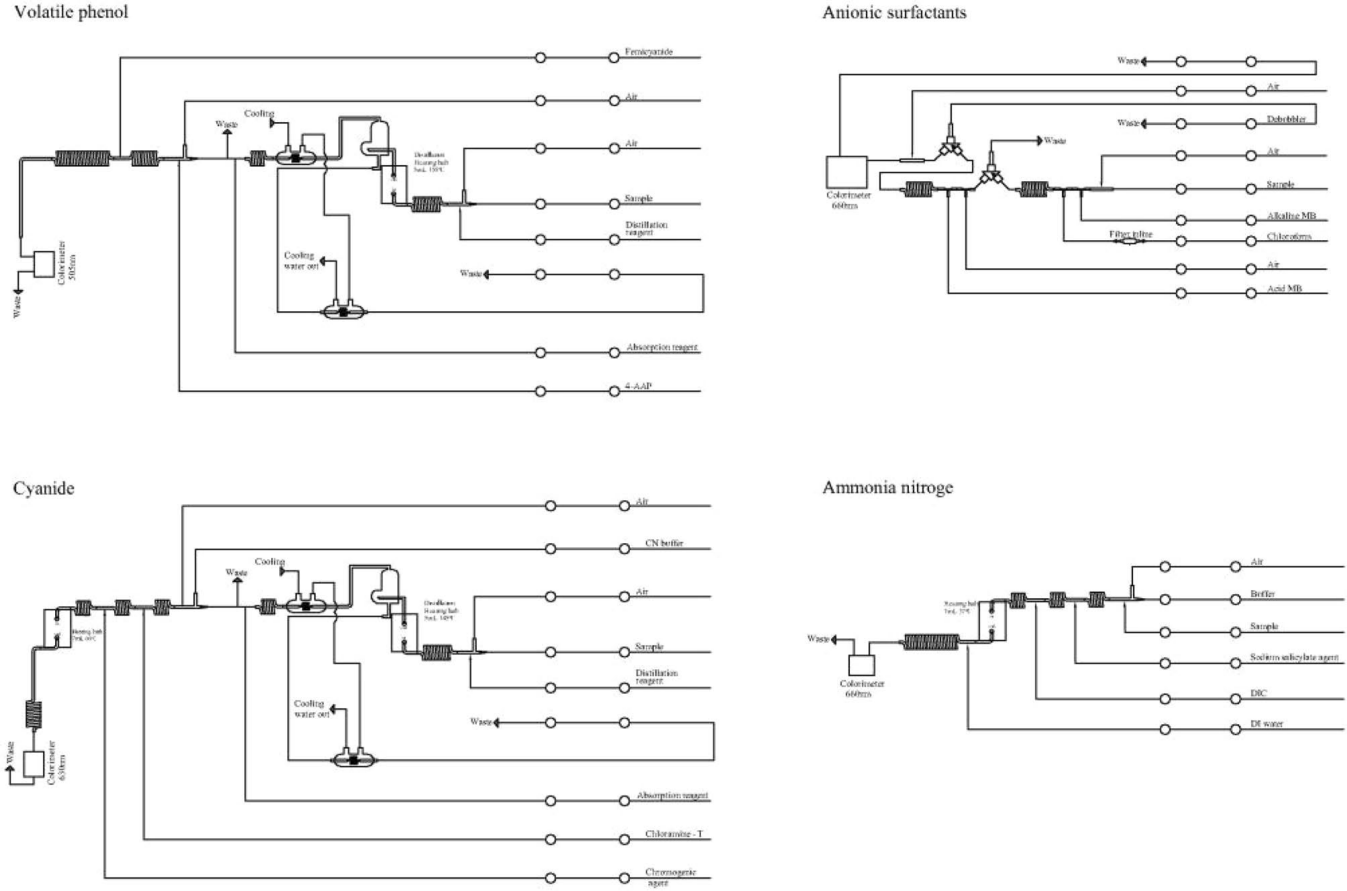
Schematic diagram for determination of volatile phenol, cyanide, anionic surfactant and ammonia nitrogen by continuous flow injection.

In this automated method for the determination of phenol and cyanide, samples were first distilled at 145 °C. Phenol in the distillate then subsequently reacted with alkaline ferricyanide and 4-aminoantipyrine to form a red complex that was measured colorimetrically at 505 nm. The cyanide in the distillate subsequently reacted with chloramine T to form cyanogen chloride, which formed a blue complex with pyridinecarboxylic acid that was measured colorimetrically at 630 nm. The anionic surfactant reacted with basic methylene blue to form a compound that was extracted into chloroform and separated by a phase separator. The chloroform phase was then washed with acidic methylene blue to remove interfering substances and separated again in a second phase separator. Blue compounds in chloroform were colorimetrically determined at 660 nm. Based on the Berthelot reaction, ammonia reacted with salicylate and chlorine from dichloroisocyanuric acid to form indophenol blue in an alkaline environment at 37 °C. Sodium nitroprusside was used as a catalyst in the reaction, and the produced color was measured at 660 nm. The principle of this method is shown in Fig. 1.

### Standard curve and detection limit

The concentration of volatile phenol and cyanide was in the range of 2–100 μg/L, and the linear correlation coefficient was 1.000, the regression equation was *y* = (3.888331E + 005)*x* + (9.938599E + 003). The correlation coefficient of cyanide was 1.000, and the regression equation was *y* = (3.551656E + 005)*x* + (9.951319E + 003). The linear relationship between anionic surfactants and ammonia nitrogen concentration was good in the range of 10–1000 μg/L. The correlation coefficients of anionic surfactants and ammonia nitrogen were 0.9995 and 0.9999, respectively. The regression equations were *y* = (2.181170E + 004)*x* + (1.144847E + 004) and *y* = (2.375085E + 004)*x* + (9.631056E + 003), respectively. The blank was continuously measured 11 times, and the detection limit of the method was obtained by dividing three times the blank standard deviation by the slope of the standard curve. The detection limits of volatile phenol, cyanide, anionic surfactants, and ammonia nitrogen were 1.2 μg/L, 0.9 μg/L, 10.7 μg/L, and 7.3 μg/L, respectively. The detection limit was lower than that of the national standard method, and the details are shown in Table 2.

**Table 2:** Standard curve, correlation coefficient and detection limit of 4 kinds of analytes.

Analyte	Line Range (µg/L)	Linear regression equation	Correlation coefficient	LOD^a^ (μg/L)	GB^b^ LOD (μg/L)
Volatile phenol	0–100	*y* = (3.888331E + 005)*x* + (9.938599E + 003)	1.0000	1.2	2.0
Cyanide	0–100	*y* = (3.551656E + 005)*x* + (9.951319E + 003)	1.0000	0.9	2.0
Anionic surfactant	0–1000	*y* = (2.181170E + 004)*x* + (1.144847E + 004)	0.9995	10.7	50
Ammonia nitrogen	0–1000	*y* = (2.375085E + 004)*x* + (9.631056E + 003)	0.9999	7.3	20

^a^LOD: Limits of detection.

^b^GB: National standard code for Chinese characters.

### Intra-day and inter-day recovery and precision

High, medium, and low-concentration standard solutions were added to the water samples that did not contain traces of the analytes. The intra-day and inter-day recoveries and precision were calculated after seven continuous measurements. As shown in Table 3, the intra-day and inter-day recovery of volatile phenol were 98.0–103.6% and 96.2–102.0%, respectively, with relative standard deviations of 0.75–2.80% and 1.27–6.10%. The intra-day and inter-day recoveries of cyanide were 101.0–102.0% and 96.0–102.4%, respectively, and the relative standard deviations were 0.36–2.26% and 2.36–5.41%. Additionally, the intra-day and inter-day recovery of anionic surfactants were 94.3–107.0% and 93.7–101.6%, with a relative standard deviation of 0.27–0.96% and 4.44–4.86%. Finally, the intra-day and inter-day recovery rates of ammonia nitrogen were 98.0–101.7% and 94.4–97.8%, with relative standard deviations of 0.33–3.13% and 4.45–5.39%, respectively, as shown in Table 3.

**Table 3:** The recoveries and relative standard deviations (RSD) at three spiked levels of 4 analytes.

Analyte	Spiked concentration (μg/L)	Intra-day recovery and precision (n = 7)			Inter-day recovery and precision (n = 7)		
		Mean value ($\overline{\mathrm{X} }$±*S*) (μg/L)	Recovery (%)	*RSD* (%)	Mean value ($\overline{\mathrm{X} }$±*S*) (μg/L)	Recovery (%)	*RSD* (%)
Volatile pheno	5	4.9 ± 0.14	98.0	2.80	4.9 ± 0.31	98.0	6.10
	25	25.3 ± 0.19	101.2	0.75	25.5 ± 0.27	102.0	1.27
	50	50.8 ± 0.49	103.6	0.96	48.1 ± 0.52	96.2	4.26
Cyanide	5	5.1 ± 0.12	102.0	2.26	4.8 ± 0.33	96.0	5.41
	25	25.3 ± 0.22	101.2	0.85	25.6 ± 0.42	102.4	2.36
	50	50.5 ± 0.18	101.0	0.36	48..8 ± 0.24	97.6	3.74
Anionic synthetic detergent	100	107.0 ± 0.29	107.0	0.27	101.6 ± 0.16	101.6	4.44
	250	235.7 ± 2.26	94.3	0.96	244.8 ± 3.11	97.9	4.61
	500	478.9 ± 2.63	95.8	0.55	468.3 ± 4.63	93.7	4.86
Ammonia nitrogen	100	98.0 ± 3.07	98.0	3.13	94.4 ± 4.17	94.4	5.35
	250	252.4 ± 2.61	101.0	1.03	244.4 ± 5.20	97.8	5.39
	500	508.3 ± 1.68	101.7	0.33	482.3 ± 5.56	96.5	4.45

### Comparison with other methods and the national standard method

Many test methods, including spectrophotometry^15–17^ and chromatography^25,26^, can be used to measure the four pollutants in water. Chemical spectrophotometry is the most recently studied method to detect these pollutants and is required by the national standard^27–31^, It requires distillation, extraction, and other steps, creating a time-consuming process with insufficient sensitivity, subpar precision, and poor accuracy. The extensive use of organic chemicals may pose health hazards to the experimenters. Although the chromatographic method is fast, simple, efficient and has low detection limit, it cannot be used to determine four compounds simultaneously. However, non-equilibrium dynamic conditions are used for chemical analysis using continuous-flow spectrophotometry, which is based on a continuous gas-interval flow of the sample solution, the addition of reagents in the proper ratio and order, the completion of the reaction while traveling through the mixing loop, and the removal of bubbles before spectrophotometric detection. As the detection process is automated, the samples are distilled and extracted online in a comparatively closed environment. This approach significantly increases the work efficiency, further reduces the detection time, streamlines the operation steps, lessens reagent pollution, and increases the method's sensitivity and detection limit.

Anionic surfactants and ammonia nitrogen were prepared into a combined test sample with a concentration of 250 μg/L. Volatile phenol and cyanide were prepared into a test sample with a concentration of 10 μg/L using standard materials. The national standard method and this method were used for analysis and detection, respectively (six parallel experiments). The outcomes of the two approaches were compared using an independent *t*-test. There was no discernible difference between the two methods (*P* > 0.05), as shown in Table 4.

**Table 4:** Comparison of determination results between national standard method and this method.

Analyte	National standard method ($\overline{\mathrm{X} }$±*S*) (μg/L)	This method ($\overline{\mathrm{X} }$±*S*) (μg/L)	*t* value	*P* value
Cyanide	10.05 ± 0.41	10.20 ± 0.24	0.781	0.453
Volatile phenol	10.15 ± 0.24	10.17 ± 0.13	0.146	0.887
Anionic surfactant	235.20 ± 3.04	235.75 ± 2.26	0.394	0.702
Ammonia nitrogen	249.83 ± 2.14	252.40 ± 2.61	1.964	0.092

## Discussion

In this study, the volatile phenol, cyanide, anionic surfactants, and ammonia nitrogen were simultaneously analyzed and detected using a continuous flow analyzer. The detection results demonstrated that the continuous flow analyzer used lower sample volumes than the national standard method. It also had a lower detection limit, used 80% less reagents, required less time to process a single sample, and used significantly less highly-carcinogenic chloroform. Online processing was integrated and automatic. Continuous flow automatically absorbed the reagent and sample, which was then blended through a mixing loop, automatically heated, extracted, and computed using colorimetry. The experimental process occurred in a closed system, which accelerated the analysis time and reduced pollution, helping ensure the safety of experimental personnel. Steps in the operation that were challenging, such as manual distillation and extraction, were unnecessary^22,32^. The instrument's pipeline and accessories, however, are complicated, and several factors may influence the test results, making it simple to cause system instability. To enhance the accuracy of results and prevent interference with the experiment, several key steps can be followed. (1) The pH of the solution should be taken into consideration when measuring volatile phenol and cyanide. The pH should be around 2 before reaching the distillation coil. If pH > 3, aromatic amines may also be distilled, and the reaction with 4-aminoantipyrine may produce errors. Additionally, the recovery rate of K_3_[Fe(CN)_6_] will be less than 90% if pH > 2.5. Samples containing more than 10 g/L of salt may cause problems by blocking the distillation coil. To lessen the sample's salt level in this situation, fresh water should be added^33^. (2) The following factors may affect the identification of anionic surfactants: Cationic chemicals may form potent ion pairs with anionic surfactants. The results may also be skewed when the following species are present: humic acids at concentrations greater than 20 mg/L; compounds with a high surface activity (such as other surfactants) > 50 mg/L; substances with strong reducing potentials (SO_3_^2-^, S_2_O_3_^2-^, and OCl^-^); substances that produce colorful molecules soluble in chloroform with any of the reagents; some inorganic anions (chloride, bromide, and nitrate) in wastewater^34,35^. (3) Small-molecule amines should be taken into consideration when calculating ammonia nitrogen since they react with ammonia similarly and will consequently produce excessively high results. If the reaction mixture's pH is below 12.6 after the addition of all reagent solutions, interference may occur. Strongly acidic and buffered samples tend to cause this. Low repeatability may also be introduced by metal ions that precipitate in high concentrations as hydroxides^36,37^.

## Conclusion

The results demonstrated that the continuous flow analysis method for the simultaneous determination of volatile phenol, cyanide, anionic surfactants, and ammonia nitrogen in drinking water had good linearity, a low detection limit, and good precision and recovery. It showed no appreciable difference from the national standard method. The approach provides a quick, sensitive, precise, and simple-to-use methodfor the analysis and determination of a large number of water samples. It is especially suitable for the simultaneous determination of four components, which results in considerably better detection efficiency.

## Data Availability

All data generated or analysed during this study are included in this published article.
